# Comparative study of robotic-assisted single-incision-plus-one port and single-incision laparoscopic choledochal cyst excision

**DOI:** 10.3389/fped.2024.1403358

**Published:** 2024-09-19

**Authors:** Ling Zhang, Shan Chen, Yang Lin, Jianbin Wang, Xinyi Qiu, Lizhi Li

**Affiliations:** ^1^ Department of Pediatric Surgery, Provincial Clinical Medical College, Fujian Medical University, Fuzhou, Fujian, China; ^2^ Department of Laboratory, Fuzhou Second General Hospital, Fuzhou, Fujian, China

**Keywords:** choledochal cyst, pediatric, hepaticojejunostomy, robotic-assisted, single-incision laparoscopic surgery

## Abstract

**Objective:**

To compare the efficacy of robotic-assisted single-incision-plus-one-port laparoscopic choledochal cyst excision (R-SILC + 1) and single-incision laparoscopic choledochal cyst (SILC) in treating pediatric choledochal cyst (CDC).

**Methods:**

We retrospectively analyzed the clinical data of patients diagnosed with CDC in our hospital from June 2021 to October 2023. Among them, patients underwent either R-SILC + 1 or SILC procedures. Demographic parameters, operative details, and postoperative outcomes were studied.

**Results:**

A total of forty-nine patients were included, with 23 children undergoing R-SILC + 1 and 26 children undergoing SILC. There were no statistically significant differences in demographic data, postoperative pain scores, and postoperative complication rates between the two groups (all *p* > 0.05). Compared with the SILC group, the R-SILC + 1 group demonstrated less intraoperative bleeding volume (10.4 ± 3.6 vs. 15.0 ± 3.6 ml, *p* < 0.05), a shorter indwelling time of the abdominal drainage tube [5(5,6) vs. 7(5.8,8.3) d, *p* < 0.05], a shorter postoperative fasting time [4(3,4) vs. 6(5,7) d, *p* < 0.05], and a shorter postoperative discharge time [6(6,7) vs. 8(6,11) d, *p* < 0.05]. However, the R-SILC + 1 group had a longer operation time [388(295,415) vs. 341(255.8,375.2) min, *p* < 0.05] and higher hospitalization cost (7.9 ± 0.4 vs. 3.2 ± 0.3 ten thousand, *p* < 0.05).

**Conclusion:**

Compared with the SILC group, the R-SILC + 1 group demonstrated clear advantages in treating pediatric CDC, but it is associated with a prolonged learning curve and operation time, and high costs. With improvements in physician experience and technological advancements, its potential will be further unleashed.

## 1 Introduction

CDC is a common disease in pediatric surgery, with abdominal pain, jaundice or abdominal mass as the main manifestations. Ultrasound typically reveals cystic or fusiform dilation of some bile ducts, which may be accompanied by intrahepatic bile duct dilation. Choledochal cysts are divided into 5 types according to Todani classification, of which type I is the most common, accounting for about 80% (1). Most choledochal cysts require surgical treatment to reduce the risk of cyst-related complications, such as cholangitis, pancreatitis, and malignant transformation (2). Farello et al. reported the first successful laparoscopic choledochal cyst excision (LC) in 1995 (3).

With advances in robotic surgery, robotic-assisted laparoscopic choledochal cyst excision (RALC) was first internationally reported for a 5-year-old girl in 2006 (4). As two emerging minimally invasive surgical methods, robotic-assisted single-incision-plus-one-port LC (R-SILC + 1) and single-incision LC (SILC) have attracted much attention due to their advantages such as small trauma, fast recovery, and cosmetic appearance. At present, there is a relative lack of comparative research on the efficacy of R-SILC + 1 and SILC. This study aimed to comparatively compare the efficacy and safety of R-SILC + 1 with SILC, in order to provide a more scientific and effective reference basis for clinical treatment.

## 2 Materials and methods

### 2.1 Clinical data

We retrospectively analyzed the clinical data from forty-nine patients diagnosed with CDC in our hospital from June 2021 to October 2023. Both surgical methods were performed by the same surgical group, and chosen by patients’ parents dependent of their preferences and economic capabilities. According to the surgical method, the patients were divided into the R-SILC + 1 group with 23 cases and the SILC group with 26 cases.

The inclusion criteria: (1) primary surgery; (2) preoperative auxiliary examination diagnosis of CDC with surgical indications; (3) normal coagulation ability, no severe cardiopulmonary dysfunction and other vital organs; (4) no deformities in other parts such as the digestive system; (5) single incision; (6) complete medical records, laboratory examination and imaging results.

The exclusion criteria: (1) secondary surgery; (2) combined intrahepatic bile duct dilation (V type in Todani classification), especially stenosis at the left and right hepatic duct; (3) cyst rupture or punching; (4) suspected degeneration of CDC; (5) combined portal vein cavernous degeneration or other structural malformations of the liver vascular system.

### 2.2 Methods

#### 2.2.1 Data collection

In addition to exclude surgical contraindications and clarify liver function,routine preoperative examinations were performed, including blood routine, C-reactive protein(CRP), urine routine, biochemical examination, coagulation function, hepatitis B virus, chest x-ray and electrocardiogram. All patients underwent ultrasonography and magnetic resonance cholangiopancreatography (MRCP) to confirm the diagnoses of CDC and Todani types. The sex, age, weight, symptoms at admission, maximum cyst diameter, Todina type and follow-up time were collected in both groups.

According to the perioperative indicators: operation time (defined as the time from umbilical incision to incision suture), intraoperative bleeding volume (calculated as the D-value of the blood-soaked and dry gauze, plus draining blood volume), postoperative abdominal drainage tube indwelling time, postoperative fasting time, and postoperative discharge time (discharge criteria: stable vital signs, restoration of normal diet, stable defecation function, and absence of incision infection or bile leakage), hospitalization cost, and postoperative pain scores (measured at 36 h using the Children's Postoperative Pain FLACDC Scale) (5). The sum of the five indicators ranges from a minimum of 0 to a maximun of 10, with higher scores indicating greater discomfort and pain). All children underwent physical examinations, ultrasonography, and biochemical examinations in outpatient clinics at 1st, 3rd, 6th, and 12th months after discharge. If necessary, abdominal computed tomography (CT) would be performed to determine the presence of long-term complications. The follow-up time for all children ranged from 4 to 33 months, with a median of 15 months.

#### 2.2.2 Surgical methods

All operations were performed by the same surgeon. On the day prior to the surgery, fasting was mandatory, and intestinal lavage should be performed. After general anesthesia, all patients were placed in the supine position with an incline of 30°-45°to the left side. The operation area was marked, a 3 cm curved incision was made around the left edge of the umbilical to accommodate a quadruple-channel single-hole laparoscopic puncture device. The surgical procedure includes extracorporeal end-to-side jejunostomy, gallbladder and choledochal cyst resection, and hepaticojejunostomy.

##### 2.2.2.1 R-SILC + 1 procedure

**Figure 1:**
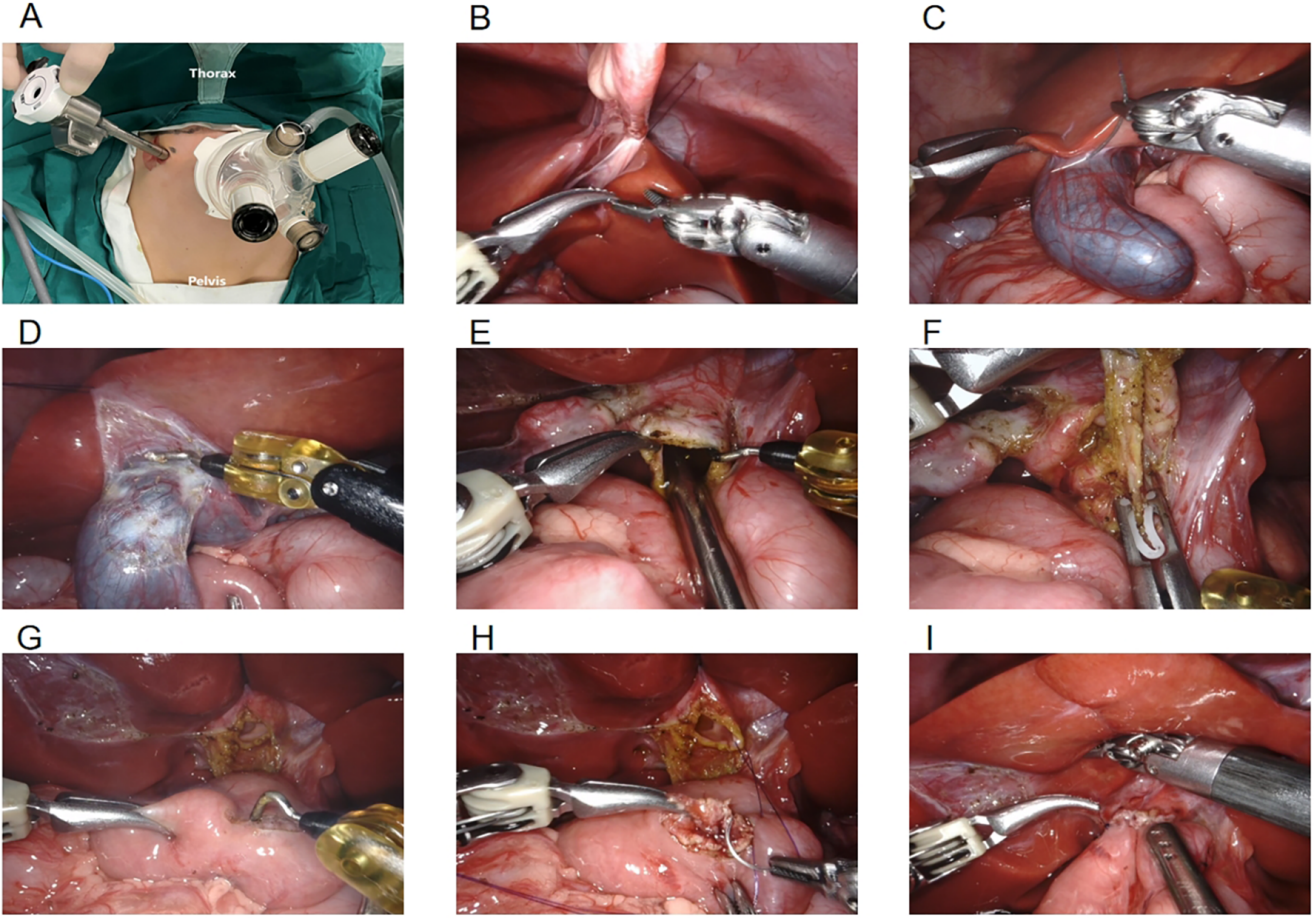
**(A)** location of robotic trocars; **(B)** suspension of the ligamentum teres hepatis; **(C)** suspension of the gallbladder serosa; **(D)** freeing the gallbladder; **(E)** sucking up bile; **(F)** clamping the distal end of the common bile duct; **(G)** incising the opposite mesentery; **(H)** common hepatic duct jejunal anastomosis; **(I)** draining the abdominal effusion.

Two 8 mm channels were inserted into a 3D camera port III and an operating port IV which can be used as an assistant port. Another 8 mm incision was made 6 cm to the right of robotic operating port II (Figure 1A).

We explored the abdominal cavity using a robotic 3D camera and laparoscopic forceps.We lifted the transverse colon to identify the Treitz ligament, grasped the jejunum 25 cm away from the ligament and exteriorized it for end-to-side anastomosis of the jejunum. After the intestine was returned to the abdominal cavity, the da Vinci system was docked.

**Figure 2:**
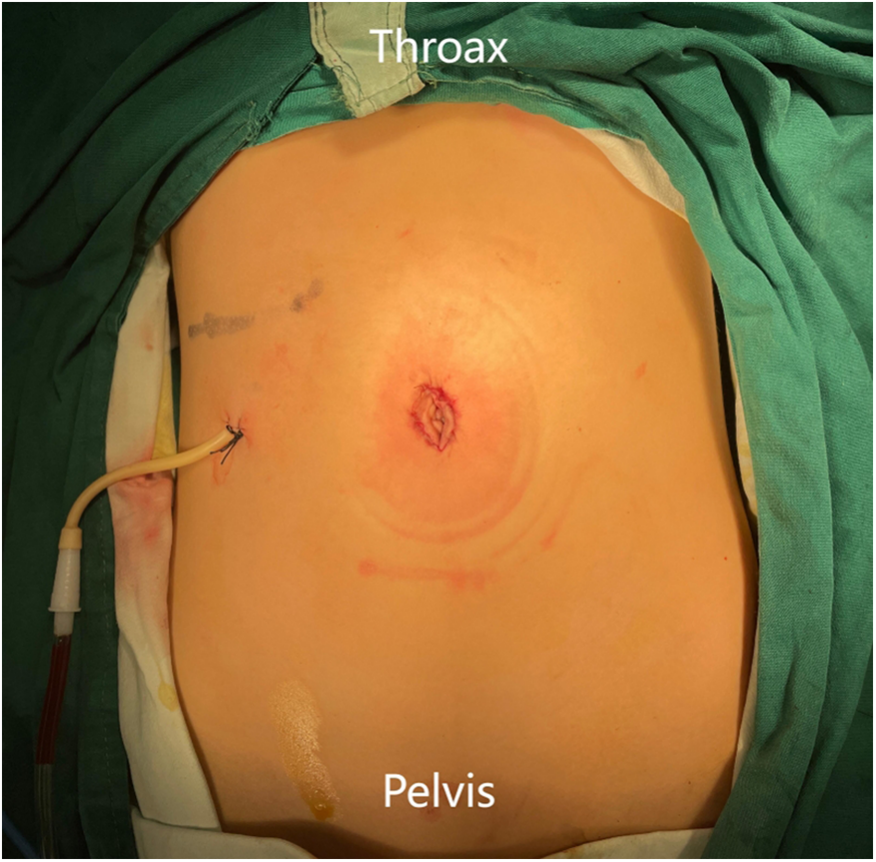
Postoperative incision.

The ligamentum teres hepatis (Figure 1B) and the gallbladder serosa (Figure 1C) were suspended to expose the hilar area by two traction sutures through the abdominal wall. The gallbladder was freed from the common bile duct (CBD) using unipolar electrocoagulation (Figure 1D).The cystic artery in Calot's triangle was detached and clamped, followed by removal of the gallbladder. The anterior wall of the cyst was incised, bile was sucked up via the the aspirator (Figure 1E) and then the cyst was transected. The forceps pulled the duodenum downward, while ultrasonic energy was used to free the cyst towards its distal end where it becomes thinner and meets the pancreatic duct. The distal end of the common hepatic duct was clamped (Figure 1F), and the distal cystic wall was removed. Horizontal resection of the common hepatic duct and removal of the proximal cyst were performed using the same method. According to the diameter of the common hepatic duct, the opposite wall of the mesentery was incised (Figure 1G). A continuous suture of the posterior wall of the common hepatic duct and the jejunum was performed using 4-0 absorbable barbed sutures (Figure 1H). The same method was used to suture the the anterior walls. The abdominal effusion was drained (Figure 1I), the puncture device was removed, and the incision was sutured closed (Figure 2) (6).

##### 2.2.2.2 SILC procedure

(1) Except for the absence of an additional lateral abdominal operating sheath, docking the robot surgical platform,and the installation and disassembly of robotic arm. (2) After laparoscopic resection of the gallbladder and choledochal cyst, end-to-side anastomosis of the jejunum was completed *in vitro*, followed by Roux-en-Y hepaticojejunostomy (7).

#### 2.2.3 Postoperative treatment

The second-generation cephalosporins were administered by intravenous infusion for 48 h, followed by oral administration of cefaclor for 3–6 days. Postoperative routine monitoring included blood routine, CRP, hepatic and renal function, electrolytes, etc. If symptoms such as fever and abdominal distension occurred, ultrasonography and hematural amylase examination were performed. Patients were fasted (besides medicine) until intestinal function was restored, which typically took 4–7 days. They were initially given water, followed by a liquid diet, and then progressed to a soft diet.

#### 2.2.4 Statistical analysis

Statistical analysis was performed using SPSS 26.0 software. The Kolmogorov-Smirnov (KS) test was used to assess whether the data conformed to a normal distribution. For normal distributed data, it was expressed as mean ± standard deviation (SD) and an independent sample *t*-test was used for comparisons between two groups. For non-normal distributed data, it was expressed as M (Q1, Q3) and the Mann-Whitney *U*-test was used for comparisons between two groups. The sample rates of the two groups were compared using χ^2^ test, and the exact χ^2^ used Fisher method. *p *< 0.05 was considered statistically significant.

## 3 Results

### 3.1 Demographic data and preoperative conditions

The demographic data and preoperative conditions patients who were diagnosed with CDC are summarized in Table 1. There were 19 males and 30 females, age ranged from 6 to 146 months, with a median of 25 months, weight ranged from 6.9 to 57.9 kilogram, with a median of 18.9 kilogram. There were 15 children accompanying with abdominal pain and 8 children with jaundice. Via Todina classification, there were 34 cases of type I and 15 cases of other types. The maximum cyst diameter was 3.3 ± 1.3 centimeter, ranging from 1.2 to 6.2 centimeter. There was no significant difference in demographic data between the two groups (*p* > 0.05).

**Table 1:** Demographic data and preoperative conditions.

Groups	Sex (%)		Age [months, M (Q1, Q3)]	Weight [kg, M (Q1, Q3)]	Symptoms (%)		The maximum cyst diameter (cm, mean ± SD)	Todina Type (%)	
	Male	Female			Yes	No		Todani I	Other types
R-SILC + 1 (*n* = 23)	8 (34.8)	15 (65.2)	27 (13,45)	20.6 (14.5,32)	11 (47.8)	12 (52.2)	3.5 ± 1.4	16 (69.6)	7 (30.4)
SILC (*n* = 26)	11 (42.3)	15 (57.7)	24 (17.3,60.8)	18.5 (12.4,26.4)	12 (46.2)	14 (53.8)	3.2 ± 1.1	18 (69.2)	8 (30.8)
t/χ2/Z	χ2 = 0.291		Z = −0.160	Z = −0.441	χ2 = 0.220		t = 0.014	χ2 = 0.001	
*P* value	0.590		0.873	0.659	0.639		0.907	0.980	

### 3.2 Intraoperative and postoperative outcomes

All procedures were successfully completed as planned and no children required conversion to laparotomy. The intraoperative bleeding volume, postoperative fasting time, postoperative abdominal drainage tube indwelling time, and postoperative discharge time of the R-SILC + 1 group were significantly less than those of the SILC group [10.4 ± 3.6 vs. 15.0 ± 3.6 ml; 4(3,4) vs. 6(5,7) d; 5(5,6) vs. 7(5.8,8.3) d; 5(5,6) vs. 7(5.8,8.3) d, respectively; *p *< 0.05]. The operation time of the R-SILC + 1 group was significantly longer than that of the SILC group [388(295,415) vs. 341(255.8,375.2) min, *p *< 0.05]. The hospitalization costs of the R-SILC + 1 group were significantly higher than that of the SILC group (7.9 ± 0.4 vs. 3.2 ± 0.3 ten thousand, *p *< 0.05). No statistically significant difference in postoperative pain scores was observed (*p = 0.134*). To date, no long-term complications have been found in children, and there was no statistically significant difference in the postoperative complication rate between the two groups (*p = *0.113) (Table 2).

**Table 2:** Perioperative and postoperative conditions.

Group	Operation time [min, M (Q1, Q3)]	Intraoperative bleeding volume[ml, mean ± SD]	Postoperative complications (%)		postoperative abdominal drainage tube indwelling time[d,M (Q1, Q3)]	postoperative pain score[point, mean ± SD]	postoperative fasting time[d,M (Q1, Q3)]	Postoperative discharge time [d, M (Q1, Q3)]	Hospitalization cost [ten thousand yuan, mean ± SD]
			Yes	No					
R-SILC + 1 (*n* = 23)	388 (295,415)	10.4 ± 3.6	1 (4.3)	22 (95.7)	5 (5,6)	1.0 ± 0.1	4 (3,4)	6 (6,7)	7.9 ± 0.4
SILC (*n* = 26)	341 (255.8,375.2)	15.0 ± 3.6	5 (19.2)	21 (80.8)	7 (5.8,8.3)	1.1 ± 0.1	6 (5,7)	8 (6,11)	3.2 ± 0.3
t/χ2/Z	Z = −2.364	t = −4.586	χ^2 ^= 2.516		Z = −3.524	t = −1.526	Z = −4.878	Z = −3.065	t = 47.595
*P* value	0.018	0.000	0.113		0.000	0.134	0.000	0.002	0.000

### 3.3 Postoperative complications and management

In the SILC group, three children exhibited postoperative abdominal infection, presenting with hyperpyreia and increased CRP levels on the 3rd day after surgery, which recovered after one week of anti-infection treatment with cefoperazone. Two children exhibited incision infection, with redness and swelling of the incision accompanied by purulent discharge on the 4th day after surgery, these were successfully treated with one week of dressing change and care. In the R-SILC + 1 group, one child exhibited peritoneal effusion, showing hyperpyreia and increased CRP levels on the 1st day after surgery, which recovered after one week of anti-infection treatment with cefoperazone.

## 4 Discussion

Both LC and RALC typically require 3 to 4 incisions. The quest to further reduce surgical trauma and the number of incisions remains a focal point in pediatric minimally invasive surgery (8). Single-incision laparoscopic surgery (SILS) has been proven to be a feasible and safe laparoscopic surgery method (9, 10). It places the surgical incision around the umbilicus and uses the periumbilical fold to achieve a beautiful postoperative scar effect (11). Cheng et al. has reported that single-incision robotic-assisted surgery (SIRAS) is safe and feasible within a limited scope of surgery, with good clinical and significant cosmetic effects (12). Since the introduction of robots to our hospital in 2020, our team has been actively exploring the application of SIRAS technology to a wider range of clinical scenarios, striving to minimize surgical trauma and maximize aesthetic effects. By continuously optimizing surgical procedures and techniques, we have applied them to the treatment of ureteropelvic junction obstruction, primary obstructed megaureters, Hirschsprung's disease, choledochal cyst, and other diseases. However, there is currently a lack of comparative research on the two surgical methods, SILC and R-SILC + 1. Building on the research of Lin et al., our study further confirms the safety and feasibility of the R-SILC + 1 surgical method.

Studies have reported that RALC is comparable to LC in postoperative efficacy (13, 14). Lin et al. has conducted a comparative study between R-SILC + 1 and SILC procedures, indicating that the total operative time for R-SILC + 1 was longer than for SILC. However, the time taken for crucial steps, such as choledochal cyst excision and mean hepaticojejunostomy anastomosis, was shorter in R-SILC + 1. Additionally, they propose that the SILS + 1 technique could overcome the limited abdominal space in pediatric patients, avoiding the drawback of robotic arms colliding during single-incision operations (6). Since the initiation of robotic surgery in our department, we have employed the SILS + 1 technique and summarized the following advantages. Firstly, the using of quadruple-channel single-hole laparoscopic puncture device allows for isolating the main operating port, placing the 2nd and 3rd arms along with the assistant instruments into the single port, and positioning the 4th arm through an additional port. It is crucial to maintain the anterior-posterior alignment of the endoscope and operating instruments during the procedure. The distance between the 2nd and 3rd arms during operation can be approximately 4–5 cm, while the distance between the 3rd and 4th arms can be around 3–4 cm. Secondly, the technique for instrument positioning and installation begins with inserting the endoscope arm into channel 2. Observing the insertion position and depth of the 4th mechanical arm under direct visualization is crucial before installing the endoscope arm and instruments for the 2nd arm, ensuring their proper installation and utilization. Thirdly, the distance between the reduced arms is crucial, especially for pediatric patients with limited body surface area. In such cases, only the 2nd, 3rd, and 4th arms are used, and through careful planning, the spacing between the operating ports is maintained at 5–6 cm to prevent collisions. Fourthly, the rational utilization of the single umbilical channel allows for the completion of intestinal anastomosis *in vitro*, thereby shortening the surgical operation time (15). Fifthly, during the procedure, sequential suspension traction is employed to traction the ligamentum teres hepatis, gallbladder serosa, CBD, and abdominal wall in the operative field. This traction ensures a full exposure of the surgical field, thereby increasing the operating space. Lastly, the reasonable use of barbed sutures during hepaticojejunostomy can reduce slippage during the suturing process, without the need for assistant instruments.

RALC, building upon the foundation of LC, significantly shortens the learning curve (16). With its stable and flexible operating system, it can filter out errors caused by hand tremors, leading to more precise cyst separation and hepatic duct anastomosis. Consequently, intraoperative suturing time is reduced compared to the LC group (6, 17). However, this study finds that the operating time for the R-SILC + 1 group was longer than that for the SILC group (*p < *0.05). RALC increases the time required for the assembly and disassembly of the robotic arms relative to LC (18). This study includes this time period in the total operating time, explaining why the results show that the operating time for RALC was longer than that for LC. Our department has extensive experience with LC procedures, while the implementation of RALC procedures is still in the early stages. In this study, the R-SILC group exhibited less blood loss compared to the SILC group (*p < *0.05). This may be attributed to robotic surgery providing 3D vision with significant magnification, enabling the clear identification of small vessels and reducing the risk of damage (19). Furthermore, the postoperative discharge time for children in the R-SILC group was shorter than that in the SILC group (*p < *0.05). This could be attributed to the more precise nature of robotic surgical procedures, resulting in minimal impact on peripheral tissues, mild inflammatory response in the surgical area, less exudation, and faster postoperative tissue repair. The above analysis indicates that the R-SILC procedure achieves similar therapeutic efficacy as SILC, while offering patients better perioperative benefits.

The SILC procedure presents significant challenges. Initially, it deviated from the traditional triangular distribution of surgical instruments, complicating the formation of an effective surgical field (20). This misalignment can lead to a phenomenon called the “coaxial effect,” causing instrument collision and interference, thereby reducing operational accuracy and posing challenges for complex surgical tasks (10). R-SILC + 1, as a combination of RALC and SILS, compensates for the “coaxial effect” and the loss of the operating triangle in SILC through the multidimensional mobility of robotic arms and specialized instruments, and has confirmed its feasibility in biliary reconstruction surgery (6, 21, 22). Despite these advances, R-SILC + 1 is not without limitations. Firstly, the surgery is associated with higher costs. In this study, hospitalization costs for the R-SILC + 1 group were significantly higher compared to the SILC group (*p < *0.05), which may limit its widespread clinical application. Secondly, the robotic surgical system lacks mechanical feedback, especially during suturing and knotting, relying solely on visual feedback for training. Additionally, the da Vinci system's inability to change the patient's position during surgery means that altering positions intraoperatively consumes a considerable amount of time (23). Lastly, the large size of the robotic system imposes restrictions when applied to young children, particularly because the existing trocars are relatively large, with an 8 mm trocar being comparatively thick for infants.

Our study is a single-center, retrospective study with a relatively small sample size, and the grouping exhibits certain selection bias. Further large-scale, multicenter, prospective studies are needed to clarify the long-term efficacy.

## 5 Conclusion

Compared with the SILC group, the R-SILC + 1 group demonstrated clear advantages in treating pediatric CDC, with significant advantages in reducing intraoperative bleeding volume, shortening postoperative fasting time, postoperative abdominal drainage tube indwelling time, and postoperative discharge time. However, it is associated with a prolonged learning curve and operation time, and high costs. With improvements in physician experience and technological advancements, its potential will be further unleashed.

## Data Availability

The original contributions presented in the study are included in the article/Supplementary Material, further inquiries can be directed to the corresponding author.
